# Absorbable Sutures and Telemedicine for Patients Undergoing Trigger Finger Release

**DOI:** 10.7759/cureus.42486

**Published:** 2023-07-26

**Authors:** S. Elliott Holbert, Cameron Brown, Samantha Baxter, Andrea H Johnson, Jeffrey Gelfand, Alexander Shushan, Justin J Turcotte, Christopher Jones

**Affiliations:** 1 Orthopedic Research, Anne Arundel Medical Center, Annapolis, USA; 2 Orthopedics, Anne Arundel Medical Center, Annapolis, USA; 3 Orthopedic Surgery, Anne Arundel Medical Center, Annapolis, USA

**Keywords:** patient satisfaction score, absorbable suture, telemedicine in orthopedics, telemedicine (tm), trigger finger release, trigger finger disorder

## Abstract

Background

In the setting of the COVID-19 pandemic, the development of care processes that reduce the need for in-person clinic visits while maintaining low complication rates is needed. The purpose of this study is to assess the outcomes of patients undergoing trigger finger release with various suture and follow-up visit types to assess the feasibility of shifting towards telemedicine-based follow-up protocols.

Methods

A retrospective review of 329 patients undergoing trigger finger release was performed. Patients were classified based on whether or not they received in-office follow-ups; whether they received absorbable or non-absorbable sutures; and whether they were treated using a telemedicine and absorbable suture protocol or other combination of sutures and follow-ups. Univariate statistics were performed to compare outcomes between groups.

Results

Patients who did not undergo in-office follow-up were more likely to experience residual stiffness or contracture (11.4% vs. 4.1%; p=0.033) but had no significant differences in 30-day reoperation, emergency department (ED) returns, wound complaints, and Quick DASH (Disabilities of the Arm, Shoulder, and Hand) scores. When comparing chromic absorbable sutures to non-absorbable sutures, those with absorbable sutures were significantly more likely to have telemedicine visits but were also more likely to have wound complaints (17.9% vs. 8.5%; p=0.022). There was no significant difference in two- and six-week pain scores, 30-day reoperation, ED returns, residual symptoms, and Quick DASH scores. When comparing patients treated using the absorbable suture and telemedicine protocol with those receiving any other type of suture and postoperative follow-up, no significant differences in any postoperative clinical outcome measures were observed.

Conclusion

The results of this study demonstrate that the use of an absorbable suture and telemedicine protocol for patients undergoing trigger finger release yields similar outcomes as traditional methods of care. However, the use of absorbable sutures may result in decreased patient satisfaction with surgical wound healing.

## Introduction

Trigger finger, also known as stenosing flexor tenosynovitis, is one of the most common causes of hand disability, with a lifetime prevalence of 2%-3% [1,2]. Trigger finger is caused by thickening of the A1 pulley or flexor tendon that disturbs the tendon-pulley interface and the condition can be treated both conservatively and surgically. Conservative management includes activity modification, immobilization, hand therapy, nonsteroidal anti-inflammatory drugs (NSAIDs), and steroid injections. Surgical management includes percutaneous or open release of the A1 pulley [1]. The open release is currently the gold standard for surgical management with a success rate between 90% and 100%, although the procedure is associated with a complication rate of 7%-43% [2]. The majority of complications are minor, including superficial infection, dehiscence, incision discomfort, and stiffness [2]. In the setting of the COVID-19 pandemic, developing and evaluating care processes that reduce the need for in-person clinic visits while maintaining low complication rates have become a priority for surgeons. In patients undergoing trigger finger release, the safety and efficacy of absorbable sutures and the feasibility of postoperative follow-up via telemedicine are key factors influencing our ability to achieve this goal.

Traditional approaches to wound closure in hand surgery primarily utilize non-absorbable sutures made from synthetic materials such as nylon, polybutester, polypropylene, or natural silk that require removal after placement [3]. Non-absorbable sutures are thought to cause less trauma and foreign body reaction, resulting in a more aesthetically pleasing scar, but carry the need for follow-up removal, which can cause significant discomfort [4,5]. In contrast, absorbable sutures encompass a variety of natural or synthetic materials, including purified bovine or sheep collagen with or without chromic salt coating, polyglycolic acid, poliglecaprone, polydioxanone, or polytrimethylene [6]. Absorbable sutures maintain their strength for 14-21 days and rely on hydrolysis to disintegrate the sutures and phagocytes to absorb the remaining particles [4,5]. Based on this property, absorbable sutures hold the potential to negate the need for routine in-person follow-up, making telemedicine follow-up a viable alternative to in-clinic visits. The elimination of unnecessary follow-up visits may lead to increased patient satisfaction and reduced costs of care [5].

The rapid expansion of telemedicine use during the COVID-19 pandemic presents an opportunity to pair this technology with absorbable sutures to realize the potential for remote follow-up after trigger finger release [7]. Telemedicine has several limitations when considering a comprehensive hand examination, which in the office setting typically includes inspection and palpation, vascular, sensory, and motor examinations. However, there are alternatives to many limitations, and a postoperative follow-up does not necessarily require a full comprehensive examination [8]. Regarding quality and safety of care, in a randomized controlled trial, Buvik et al. compared 400 orthopedic telemedicine visits to traditional office visits and found the two were equivalent with respect to adverse safety events [9]. In a separate study on patient satisfaction, Buvik et al. reported no differences in patient satisfaction between telemedicine visits and in-person visits. Additionally, they found that 86% of patients preferred a telemedicine visit as their next consultation [10].

The purpose of this study was to assess the outcomes of patients undergoing trigger finger release with non-absorbable versus absorbable sutures and of those undergoing in-office versus no in-office follow-up. Based on these findings, we aim to assess the feasibility of an absorbable suture with a telemedicine follow-up protocol. We hypothesize that both absorbable sutures and telemedicine will yield similar outcomes as the traditional wound closure and follow-up approaches.

## Materials and methods

This study was deemed institutional review board exempt by the institutional clinical research committee as a review of existing electronic medical records and previously collected data. A retrospective chart review of all patients undergoing trigger finger release by three board-certified surgeons at a single institution was performed. The timeline for inclusion was between July 1, 2019 and June 30, 2021. All patients undergoing surgery during this time period were included in the study; patients who underwent concomitant procedures were excluded. No other inclusion or exclusion criteria were used. The study time period was selected to coincide with the increased use of telemedicine and absorbable sutures during the COVID-19 pandemic. A manual chart review was performed to assess patient outcomes and utilization patterns.

Perioperative protocol

Prior to the incision, digital blocks were performed on the operative finger(s) using local anesthetic. Following this, the Esmarch bandage was used to exsanguinate the limb, and the tourniquet was elevated to 250 mmHg. A standard incision overlying the A1 pulley was then undertaken down through the skin and subcutaneous tissues. Blunt dissection was carried down to the level of the flexor sheath. The A1 pulley was identified and transected perpendicular to its fibers all the way down to the A2 pulley. Once this was completed under direct vision, the wound was copiously irrigated, and the tourniquet was released. Wounds were closed using 4-0 chromic absorbable or nylon non-absorbable suture in an interrupted fashion and covered with dry sterile dressings. Suture choice was at the discretion of the surgeon at the time of surgery based on a number of patient and facility factors. Patients were discharged with oral narcotics as needed for pain control and encouraged to elevate the hand and move fingers as allowed by the bandage. Patients receiving absorbable sutures were instructed to remove the bandage five days postoperatively, shower with the wound uncovered, and replace it with a dry adhesive bandage. In-office follow-up for suture removal was scheduled at 10-14 days postoperatively, with a six-week repeat follow-up scheduled as needed. Patients receiving absorbable sutures followed the same postoperative protocol but were given the option of having the 10- to 14-day postoperative wound check via telemedicine. Patients were instructed to return to the office for suture removal if they were still present four weeks postoperatively.

Study population

A total of 329 patients underwent trigger finger release over the study period and were analyzed. Patients were classified by whether they had an in-office follow-up visit (n=241) or no in-office follow-up (n=88) and whether they used telemedicine for follow-up care (yes, n=107; no, n=221). Patients were also classified based on whether they received chromic absorbable (n=106) or non-absorbable sutures (n=223). Using these variables, patients were then classified based on whether they were treated using the absorbable suture and telemedicine protocol (defined as chromic absorbable sutures, telemedicine follow-up, and no in-office follow-up). Fifty-six patients (17%) were treated using this approach, while 273 (83%) were not.

Study outcomes

Postoperative outcomes of interest included a two-week follow-up and pain score, six-week follow-up and pain score, 30-day reoperation, and a 30-day emergency department (ED) return. The number of follow-up visits and rates of wound complaints and residual symptoms were assessed over the six-week postoperative period. Wound complaints and residual symptoms were further classified by the specific type of issue encountered. Patient-reported outcomes during the postoperative period were evaluated using the Quick DASH (Disabilities of the Arm, Shoulder, and Hand) instrument.

Statistical analysis

Based on a priori power analysis, 51 patients per group were deemed necessary to detect a medium effect size (d=0.5) between continuous endpoints evaluated using two-sided independent samples t-tests at α=0.05 and 80% power. For categorical endpoints, 44 patients per group were needed to detect a medium effect size (w=0.3) using the chi-square test at α=0.05 and 80% power. Therefore, the study was deemed adequately powered to detect medium effects when comparing both suture types and types of follow-up visits. Statistical analyses were used to determine the impact of office visits, absorbable sutures, and utilization of the absorbable suture and telemedicine protocol on patient outcomes. Univariate analysis, including chi-square tests and two-sided independent samples t-tests, was used to determine differences between groups. Fisher’s exact test was performed when the assumptions of chi-square testing were not met. All statistical analyses were performed using R Studio (Version 1.4.1717© 2009-2021, RStudio, PBC). Statistical significance was assessed at p<0.05.

## Results

Of the 329 patients, 106 (32.2%) received chromic absorbable sutures. For the two-week follow-up, 69.0% did so in-office, 26.1% did so via telemedicine and 4.9% did not follow-up. For the six-week follow-up, 23.7% did so in-office, 14.6% did so via telemedicine, and 61.7% did not follow-up. In total, there were 499 follow-up visits, 70.3% in-office and 29.7% via telemedicine. Overall, the mean time to the last follow-up was 34.0 days (Table 1).

**Table 1:** Utilization patterns. All data are presented as n (%) or mean±SD.

Utilization patterns	All patients (n=329)
Chromic absorbable sutures	106 (32.2)
Two-week follow-up	
None	16 (4.9)
In-office	227 (69.0)
Telemedicine	86 (26.1)
Six-week follow-up	
None	203 (61.7)
In-office	78 (23.7)
Telemedicine	48 (14.6)
Number of postoperative follow-ups	
Total	499
In-office	351 (70.3)
Telemedicine	148 (29.7)
Time to follow-up (days)	33.96±38.38

When comparing in-office to no in-office follow-ups, those receiving in-office care had a significantly higher two-week follow-up pain score (2.0±2.0 vs. 0.7±0.6; p=0.029) and six-week follow-up pain score (1.4±1.7 vs. 0.00±0.00; p<0.001). Those who did not undergo in-office follow-up had a higher proportion of patients with residual stiffness or contracture (11.4% vs. 4.1%; p=0.033) and a greater number of follow-up visits (1.4±0.7 vs. 1.2±0.7; p=0.028). There was no significant difference in 30-day reoperation, ED returns, wound complaints, and Quick DASH scores between those who followed up in-office and those who did not (Table 2).

**Table 2:** Patient outcomes of no in-office versus in-office follow-up. ED: emergency department; DASH: Disability of the Arm, Shoulder, and Hand. p-values <0.05 in bold. Data are expressed as mean±SD or n (%). ^*^Fisher’s exact test. ^a^n=15; ^b^n= 59.

Outcome	No in-office follow-up (n=88)	In-office follow-up (n=241)	p-value
Two-week follow-up			<0.001*
None	13 (14.8)	3 (1.2)	
In-office	0 (0)	227 (94.2)	
Telemedicine	75 (85.2)	11 (4.6)	
Two-week pain score	0.7±0.6	2.0±2.0	0.029
Six-week follow-up			<0.001*
None	58 (65.9)	145 (60.2)	
In-office	0 (0)	78 (32.4)	
Telemedicine	30 (34.1)	18 (7.5)	
Six-week pain score	0.0±0.0	1.4±1.7	<0.001
30-Day reoperation	6 (6.8)	8 (3.2)	0.214
ED return	6 (6.8)	13 (5.4)	0.837
Wound complaint	11 (12.5)	27 (11.2)	0.896
Dehiscence	0 (0)	4 (1.7)	0.577*
Superficial surgical site infection	2 (2.3)	1 (0.4)	0.176*
Tenderness/inflammation	6 (6.8)	17 (7.1)	1.000*
Thickness/eschar/erythema	3 (3.4)	3 (1.2)	0.196
Other reaction	0 (0)	2 (0.8)	1.000*
Residual symptoms	14 (15.9)	40 (16.6)	1.000
Allergic reaction	0 (0)	2 (0.8)	1.000*
Locking/clocking	0 (0)	6 (2.5)	0.348*
Pain/swelling	2 (2.3)	11 (4.6)	0.526*
Paresthesia	2 (2.3)	10 (4.1)	0.526*
Stiffness/contracture	10 (11.4)	10 (4.1)	0.033*
Weakness	0 (0)	1 (0.4)	1.000*
Quick DASH	35.2±23.8^a^	31.2±24.3^b^	0.589
Quick DASH months	5.5±4.8^a^	7.5±8.2^b^	0.232
Follow-up visits	1.4±0.7	1.2±0.7	0.028

When comparing chromic absorbable sutures to non-absorbable sutures, those with chromic absorbable sutures were significantly more likely to have a telemedicine visit at two weeks (63.2% vs. 8.5%; p<0.001) and six weeks postoperatively (28.3% vs. 8.1%; p<0.001). In addition, the chromic absorbable suture group was more likely to have wound complaints (17.9% vs. 8.5%; p=0.022). There was no significant difference in two- and six-week pain scores, 30-day reoperation, ED returns, residual symptoms, Quick DASH scores, and number of follow-up visits between those who had chromic absorbable sutures and those who had non-absorbable sutures (Table 3).

**Table 3:** Patient outcomes of chromic absorbable sutures versus non-absorbable sutures. ED: emergency department; DASH: Disabilities of the Arm, Shoulder, and Hand. p-values <0.05 in bold. Data are expressed as mean±SD or n (%). ^*^Fisher’s exact test. ^a^n=28; ^b^n=46.

Outcome	Chromic absorbable suture (n=106)	Non-absorbable suture (n=223)	p-value
Two-week follow-up			<0.001*
None	8 (7.6)	8 (3.6)	
In-office	31 (29.2)	196 (87.9)	
Telemedicine	67 (63.2)	19 (8.5)	
Two-week pain score	1.6±2.9	2.0±1.9	0.661
Six-week follow-up			<0.001*
None	57 (53.8)	146 (65.4)	
In-office	19 (17.9)	59 (26.5)	
Telemedicine	30 (28.3)	18 (8.1)	
Six-week pain score	1.2±1.7	1.3±1.8	0.870
30-Day reoperation	4 (3.8)	10 (4.5)	1
ED return	4 (3.8)	15 (6.7)	0.402
Wound complaint	19 (17.9)	19 (8.5)	0.022
Dehiscence	1 (0.9)	3 (1.3)	1.000*
Superficial surgical site infection	1 (0.9)	2 (0.9)	1.000*
Tenderness/inflammation	11 (10.4)	12 (5.4)	0.108*
Thickness/eschar/erythema	5 (4.7)	1 (0.4)	0.014*
Other reaction	1 (0.9)	1 (0.5)	0.541*
Residual symptoms	24 (22.6)	30 (13.5)	0.054
Allergic reaction	0 (0)	2 (0.9)	1.000*
Locking/clocking	2 (1.9)	4 (1.8)	1.000*
Pain/swelling	5 (4.7)	8 (3.6)	0.763*
Paresthesia	6 (5.6)	6 (2.7)	0.212*
Stiffness/contracture	10 (9.4)	10 (4.5)	0.088*
Weakness	1 (0.9)	0 (0)	0.322*
Quick DASH	33.75±26.86^a^	30.90±22.02^b^	0.643
Quick DASH months	4.38±4.50^a ^	8.80±8.70^b^	0.006
Follow-up visits	1.70±0.65	1.55±0.85	0.218

Finally, when comparing patients treated using the absorbable suture and telemedicine protocol with those receiving other combinations of suture type and postoperative follow-up, no significant differences in any postoperative clinical outcome measures, including two- and six-week pain scores, reoperation rates, ED returns, wound complaints, residual symptoms, and Quick DASH scores, were observed (Table 4).

**Table 4:** Patient outcomes of the absorbable suture and telemedicine protocol versus other suture/follow-up combinations. ED: emergency department; DASH: Disabilities of the Arm, Shoulder, and Hand. p-values <0.05 in bold. Data are expressed as mean±SD or n (%). ^*^Fisher’s exact test. ^a^n=6; ^b^n=66.

Outcome	Absorbable suture and telemedicine protocol (n=56)	Other suture/follow-up combination (n=273)	p-value
Two-week follow-up			<0.001
None	0 (0.0)	16 (5.9)	
In-office	0 (0.0)	227 (83.1)	
Telemedicine	56 (100.0)	30 (11.0)	
Two-week pain score	0.7±0.6	2.0±2.0	0.259
Six-week follow-up			<0.001
None	30 (53.6)	173 (63.4)	
In-office	0 (0.0)	77 (28.2)	
Telemedicine	26 (46.4)	23 (8.4)	
Six-week pain score	0.0±0.0	1.4±1.7	0.276
30-Day reoperation	3 (5.4)	11 (4.0)	0.654
ED return	3 (5.4)	16 (5.9)	0.883
Wound complaint	7 (12.5)	31 (11.4)	0.807
Dehiscence	0 (0.0)	4 (1.5)	1.000*
Superficial surgical site infection	1 (1.8)	2 (0.7)	0.430*
Tenderness/inflammation	4 (7.1)	19 (7.0)	1.000*
Thickness/eschar/erythema	2 (3.6)	4 (1.5)	0.271*
Other reaction	0 (0.0)	2 (0.7)	1.000*
Residual symptoms	9 (16.1)	45 (16.5)	0.931
Allergic reaction	0 (0.0)	2 (0.7)	1.000*
Locking/clocking	0 (0.0)	6 (2.2)	0.595*
Pain/swelling	1 (1.8)	12 (4.4)	0.705*
Paresthesia	2 (3.6)	10 (3.7)	1.000*
Stiffness/contracture	6 (10.7)	14 (5.1)	0.125*
Weakness	0 (0.0)	1 (0.4)	1.000*
Quick DASH	43.9±26.1^a^	30.9±23.5^b^	0.200
Quick DASH months	8.0±5.3	7.1±7.9	0.779
Follow-up visits	29.6±23.3	34.9±40.7	0.354

## Discussion

The results of this study demonstrate that the use of an absorbable suture and telemedicine protocol for patients undergoing trigger finger release appears to yield similar outcomes as traditional methods of care. However, the use of absorbable sutures may result in decreased satisfaction with surgical wound healing. In this study of 329 patients, compared to those that received non-absorbable sutures, patients who received absorbable sutures were more likely to have wound complaints, although they had similar rates of postoperative complications. Patients who received absorbable sutures were also more likely to utilize telemedicine visits at two- and six-week follow-ups. Based on these results, we suggest telemedicine and absorbable sutures are viable alternatives to traditional in-clinic follow-ups and the use of traditional sutures in the trigger finger release population, but surgeons should be prepared to address decreased patient satisfaction with postoperative wound healing when using absorbable sutures. This will be accomplished with increased patient education and informed decision-making.

Our findings regarding the results of using absorbable sutures are similar to other studies in the literature. In a randomized controlled trial comparing nylon and chromic gut sutures in minor hand surgery, Kortlever et al. found that there were more wound issues among patients receiving the chromic suture, although overall satisfaction and satisfaction with wound appearance were similar among groups [4]. Additionally, in a prospective study comparing absorbable and non-absorbable sutures used in the closure of hand surgery, Kundra et al. found no differences in wound satisfaction or Quick DASH scores among patients at two-week, six-week, and three-month follow-up [11]. These results, along with our own, suggest that surgeons can expect similar rates of postoperative complications utilizing either suture type, although patients receiving absorbable sutures may have a greater number of complaints. 

In the setting of COVID-19, the use of telemedicine in clinical practice has expanded significantly. Within orthopedics, recent studies have found that when compared to those receiving in-office visits, those who received telemedicine visits had similar rates of satisfaction, decreased travel costs and time, and additionally, most patients preferred telemedicine for their next visit [7]. Regarding the effectiveness of follow-up telemedicine visits, in a previous randomized controlled trial of video-assisted orthopedic telemedicine visits, physicians rated their ability to examine the patient as good or very good for 98% of visits [9]. To quell concerns over performing an adequate follow-up exam, Tofte et al. reported that 94% of patients were able to take adequate smartphone photos of their wounds following carpal tunnel surgery [12]. Considering our study, the utilization of telemedicine gave patients the possibility to follow up on their surgery without having to return to the office, effectively saving them time and the cost of travel in addition to helping them avoid unnecessary potential exposure to COVID-19. Notably, we found that regardless of suture type or initial visit type, the majority of patients (61%) did not return in any capacity for the six-week follow-up. This finding suggests that the six-week follow-up visit may be unnecessary for most patients and may be considered on an as-needed basis.

While multiple studies have demonstrated that telemedicine visits can be effectively performed and result in high levels of patient satisfaction in the orthopedic setting [13,14], a consensus regarding the economic impact of the approach has not been reached. A systematic review of nine studies evaluating the cost impacts of telemedicine for orthopedic care found wide variability in the level of savings achieved by using telemedicine, ranging from $10.31 to $1,755 per patient, although studies were heterogeneous in the methodologies used to evaluate cost [14]. In another randomized study comparing 199 patients receiving telemedicine and 190 patients receiving standard orthopedic consultations, the use of telemedicine was found to be cost-effective as long as a minimum of 151 consultations were performed per year. For an annual workload of 300 consultations, a cost savings of €18,616 was identified [15]. Conflicting evidence suggests that the incorporation of telemedicine may negatively impact the financial performance of high-volume orthopedic practices. In a study of academic orthopedic practice with 140,000 annual visits, the use of telemedicine for 7% of visits resulted in a negative impact of -0.8% on the institution's bottom-line financial performance [16]. This impact was projected to increase to -1.8% and -3.3% with adoption rates of 15% and 30%, respectively, driven by decreased revenue and increased expenses from technology investment and incremental staffing required to support telemedicine visits [16]. Based on the studies presented, while telemedicine does appear to hold the potential to yield cost savings in the orthopedic setting, an overarching strategy for its use across multiple subspecialties is needed to achieve economies of scale and mitigate the risk of negative financial implications. Our results showing the safety of telemedicine follow-ups in trigger finger release patients suggest this population is appropriate for incorporation into a broader orthopedic telemedicine program.

This study is not without limitations. The study was conducted at a single institution, and therefore, the results may not be applicable to the general population. In addition, as an observational study, the potential for selection bias exists. Patients may have had previous experience with telemedicine, enticing them to choose one type of visit over another. In addition, our comparison of outcomes between patients treated using the telemedicine and absorbable suture protocol is limited by the fact that patients may have deviated from the protocol and scheduled in-office visits if they experienced complications or had significant wound concerns. Finally, it is possible that other unmeasured confounding factors aside from the type of suture used and the type of postoperative follow-up visit influenced our results.

Despite these limitations, our results demonstrate the success of utilizing absorbable sutures and the effectiveness of incorporating telemedicine follow-up visits in this patient population. Surgeons should be aware that patients receiving absorbable sutures may be more likely to have wound complaints at follow-up visits and should therefore choose the suture type at their own discretion.

## Conclusions

The use of an absorbable suture and telemedicine protocol for patients undergoing trigger finger release appears to yield similar outcomes as traditional methods of care. However, the use of absorbable sutures may result in decreased satisfaction with surgical wound healing. Based on these results, we suggest telemedicine and absorbable sutures are viable alternatives to traditional in-clinic follow-ups and the use of traditional sutures in the trigger finger release population, but surgeons should be prepared to address decreased patient satisfaction with postoperative wound healing when using absorbable sutures.
